# Maternal Perceived Stress During the COVID-19 Pandemic: Pre-Existing Risk Factors and Concurrent Correlates in New York City Women

**DOI:** 10.3389/ijph.2022.1604497

**Published:** 2022-04-11

**Authors:** Akhgar Ghassabian, Melanie H. Jacobson, Linda G. Kahn, Sara G. Brubaker, Shilpi S. Mehta-Lee, Leonardo Trasande

**Affiliations:** ^1^ Department of Pediatrics, New York University Grossman School of Medicine, New York, NY, United States; ^2^ Department of Population Health, New York University Grossman School of Medicine, New York, NY, United States; ^3^ Department of Environmental Medicine, New York University Grossman School of Medicine, New York, NY, United States; ^4^ Department of Obstetrics and Gynecology, New York University Grossman School of Medicine, New York, NY, United States

**Keywords:** pregnancy, COVID-19, population health, maternal stress, post-birth

## Abstract

**Objective:** We examined whether pre-pandemic mental health and sociodemographic characteristics increased the susceptibility of pregnant women and mothers of young children to stress in the early months of the COVID-19 pandemic.

**Methods:** Between April and August 2020, we surveyed 1560 women participating in a sociodemographically diverse birth cohort in New York City. Women reported their perceived stress, resiliency, and financial, familial/societal, and health-related concerns. We extracted pre-pandemic information from questionnaires and electronic health records.

**Results:** Pre-pandemic history of depression, current financial difficulties, and COVID-19 infection were the main risk factors associated with high perceived stress. Being Hispanic and having higher resiliency scores and preexisting social support were protective against high perceived stress. Major contributors to current perceived stress were financial and familial/societal factors related to the COVID-19 pandemic. Among pregnant women, changes to prenatal care were common, as were changes to experiences following birth among postpartum women and difficulties in arranging childcare among mothers of young children.

**Conclusion:** Our findings suggest that major risk factors of higher stress during the pandemic were similar to those of other major traumatic events.

## Introduction

Nearly 2 years into the Coronavirus Disease 2019 (COVID-19) pandemic, our understanding of the presentation, management, and prevention of the disease has improved substantially. Knowledge is also increasing regarding the pandemic’s immediate and medium-term mental health outcomes, particularly in pregnant women and mothers of young children, who have been disproportionately affected [1, 2]. With the outbreak of COVID-19 in early 2020, pregnant women experienced disruption of their routine prenatal care, with increasing anxiety about potential COVID-19 infection during labor [3]. Early reports also suggested worse pregnancy outcomes, such as maternal deaths, stillbirth, and maternal depression during the pandemic compared to pre-pandemic periods [4]. These health-related concerns contributed to increased stress in pregnant women [5]. Stay-at-home orders and school closures led to heightened parental stress and reduced mental health, which disproportionately affected mothers [6]. Emotional distress in parents was highest among parents of children enrolled in remote learning versus parents of children enrolled in in-person schooling [7]. Mothers were substantially more engaged than fathers in childcare and schooling during the pandemic [8, 9], adding to the heightened stress among women with young children [10].

Perceived stress, particularly in the context of traumatic events, is an important predictor of poor mental health outcomes [11, 12] and explains a potential pathway through which stressful events lead to mental health problems or exacerbate preexisting ones [13]. While it is typical for many to report reduction in stress shortly after a traumatic event, with very few continuing on to develop long-term psychopathology or disability, the impact of the COVID-19 pandemic may pose persistent and potentially more severe risks, specifically in women [14]. Early data related to the COVID-19 pandemic suggest that individual characteristics are important predictors of mental health outcomes [15, 16]; however, most of our knowledge is based on cross-sectional surveys, which might be biased by inaccurate recall of pre-pandemic circumstances [17, 18]. Women experienced a mass exodus from the workforce and increased demands from familial obligations [19], which might have long-term consequences for mental health. It follows that there may also be persistent effects on their children due to their vulnerable life stage and the potential influence of maternal psychopathology on child health and development [20].

In this study, we address the question whether pre-pandemic mental health and sociodemographic characteristics might increase susceptibility to stress in women during the pandemic. We used existing pre-pandemic data from a well-characterized cohort in New York City (NYC) and combined it with assessment of perceived stress during the early stage of the pandemic. We examined the extent to which preexisting sociodemographic and psychosocial risk factors of stress were associated with current stress in pregnant women and/or mothers of young children. We further identified concurrent correlates and major antecedent contributors to COVID-19-related stress in these women.

## Methods

This study used data from the New York University Children’s Health and Environment Study (NYU CHES, 2016-present), an ongoing birth cohort enrolling women in early pregnancy. Women are recruited at NYU-affiliated prenatal care sites in Manhattan and Brooklyn and followed through childbirth. Mothers and children are enrolled in the postnatal phase following a live birth. Characteristics of women participating in NYU CHES are described elsewhere [21]. Between April and August 2020, in response to the COVID-19 pandemic, we surveyed participants of NYU CHES who either had a live birth or were still pregnant (*n* = 2603). Pregnant women or mothers of young children reported on their perceived stress and financial, familial/societal, and health-related concerns during the early stage of the pandemic. Surveys were administered in English, Spanish, or Mandarin, and participants filled in questionnaires online or via phone interviews. The survey contained questions on COVID-19 symptoms and diagnoses, pre-COVID and current financial stability, and pre-COVID and current stress. Questionnaire administration closed on 31 August 2020.

All participants provided written informed consent and the Institutional Review Board of the NYU Grossman School of Medicine approved the study. The authors assert that all procedures contributing to this work comply with the ethical standards of the relevant national and institutional committees on human experimentation and with the Helsinki Declaration of 1975, as revised in 2008.

### Pre-Pandemic Assessments

In NYU CHES, questionnaires administered in pregnancy, at birth, and at periodic intervals throughout infancy and early childhood collect sociodemographic information, e.g., race/ethnicity, education, employment status, and household income, medical history, and other experiences of mothers and children, including assessments of mental health and social support. Maternal depressive symptoms are assessed using the Patient Health Questionnaire (PHQ)-9 in each trimester of pregnancy, with scores ≥10 indicating depression [22]. Depressive symptoms after pregnancy are assessed using the Edinburgh Postnatal Depression Scale (EPDS) at four, eight, and 12 months after delivery, with scores ≥10 indicating postpartum depression [23]. Both PHQ-9 and EPDS are validated tools recommended by the American College of Obstetricians and Gynecologists for screening of depression in the perinatal period [24]. A composite “ever depressed” measure was derived using a combination of PHQ-9 and EPDS assessments. Social support is measured mid-pregnancy using the 7-item ENRICHD Social Support Instrument [25]; we omit the question on marital status, as it is assessed separately [26].

### Stressors During the Pandemic

In the COVID-19 questionnaire, women were asked to retrospectively report their pre-pandemic stress using a scale of 1–10, which was adapted from the American Psychological Association’s Stress in America survey [27]. Current stress was assessed using the validated 4-item Perceived Stress Scale [28]. Resilience was measured using the Brief Resilience Scale [29]. Pre-COVID and current financial security were assessed using a question adapted from the Established Populations for the Epidemiologic Study of the Elderly studies, which measured a subjective appraisal of household resources [30, 31]. Lastly, women were asked about their level of concern about 19 COVID-19-related scenarios (Supplementary Table S1). Women were also asked if they had a young child enrolled in childcare, daycare, or preschool before the pandemic and, if so, whether they experienced several types of disruptions, such as daycare or school closure.

### Statistical Analysis

The primary outcome was current PSS-4 score and represented a subjective measure of perceived stress. For bivariate analysis, PSS-4 score was considered in three categories according to the lowest quartile (low stress, PSS-4 score = 0–4), the interquartile range (moderate stress, PSS-4 score = 5–8), and the highest quartile (high stress, PSS-4 score = 9–16). Participant characteristics were assessed across PSS-4 score categories and ANOVA or t-tests were used to test for statistical differences. Multivariable linear regression models were fit to estimate the mutually adjusted associations between participant characteristics and continuous PSS-4 scores. Variables were selected *a priori* and based on the existing literature of factors associated with stress, such as age and other sociodemographic factors, as well as COVID-specific and health-related concerns [32–34]. Models yielded beta coefficients (*β*) representing the average change in PSS-4 score per unit change in each exposure and 95% confidence intervals (CI). To account for potential clustering within study sites, we reran the regression models using generalized estimating equations (GEE). In a sensitivity analysis, we excluded women who took the questionnaire after 1 June 2020, when the lockdown lifted in NYC, and those residing outside the NYC metropolitan area. To examine whether the associations between participant characteristics and current perceived stress varied by baseline (i.e., pre-COVID) financial security, the model was fit again stratifying by baseline financial security (comfortable with extra; enough but no extra; and have to cut back or cannot make ends meet).

COVID-19-related concerns were also considered in relation to current perceived stress. First, the 19 concerns were classified into three domains based on subject matter: financial, health-related, and familial/societal concerns (Supplementary Table S1). We examined internal consistency within domains using Cronbach’s alpha (standardized *α* = 0.91, *α* = 0.90, and *α* = 0.78 for the financial, health, and familial/societal domains, respectively). Average scores within each domain were derived by assigning the following: 0 = not concerned at all; 1 = slightly concerned; 2 = somewhat concerned; 3 = moderately concerned; 4 = extremely concerned. The scores for each question in each domain were summed and divided by the number of questions answered, yielding an average concern score within each domain (range = 0–4). These concern scores were simultaneously added to the above-described multivariable linear regression model with continuous PSS-4 score as the outcome.

Finally, changes to prenatal care, birth plans, postnatal care/experiences, and childcare or preschool were examined across women who were currently pregnant (*n* = 228), recently postpartum (i.e., had given birth since 1 March 2020) (*n* = 103), and with young children enrolled in childcare, daycare, or preschool before the pandemic (*n* = 531).

## Results

A total of 1560 participants (60%) completed the COVID-19 questionnaire by 31 August 2020. Respondents were generally similar to those who did not respond to the COVID-19 survey in terms of age, employment, and education (Table 1). Those who responded were more likely be partnered (88.8% vs. 85.5%) and from our Brooklyn site, which serves a low-income, majority Hispanic population (35.4% vs. 28.1%). Also, this study sample was similar to the underlying cohort in terms of sociodemographic characteristics [21]. The mean PSS-4 score among respondents of the COVID-19 survey was 6.2 (standard deviation = 2.8). Supplementary Tables S2, S3 summarize participant characteristics and bivariate associations between these characteristics and current perceived stress, suggesting that perceived stress varied by participant characteristics.

**TABLE 1:** Demographic profiles of New York University Children’s Health and Environment Study COVID-19 survey respondents and non-respondents (New York City, United States, 2020).

	COVID-19 survey respondents *n* = 1560, 59.9%	CHES participants who did not respond to the survey *n* = 1046, 40.1%
Age, mean (SD)	32.1 (5.6)	31.8 (5.7)
Marital Status, % (*n*)		
Single	11.2 (170)	14.5 (144)
Partnered	88.8 (1354)	85.5 (846)
Race/ethnicity, % (*n*)		
Hispanic	50.2 (781)	42.9 (441)
White	33.3 (518)	33.0 (381)
Black	4.5 (70)	6.7 (69)
Asian	9.1 (141)	9.8 (101)
Other/Multiple	3.0 [46]	3.6 [37]
Insurance status, % (*n*)		
Public	50.3 (770)	45.9 (470)
Private	49.7 (761)	54.1 (553)
Employment Status, % (*n*)		
No	34.7 (527)	33.4 (331)
Yes	65.3 (992)	66.6 (659)
Annual household income, % (*n*)		
<$30,000	18.1 (272)	14.8 (130)
$30,000 to $100,000	18.0 (270)	20.5 (180)
≥$100,000	40.0 (601)	42.0 (369)
Don’t know	23.9 (360)	22.7 (199)
Education, % (*n*)		
High school or less	33.1 (504)	29.6 (268)
Some college	14.6 (223)	17.7 (160)
Bachelor’s degree	24.0 (365)	24.6 (222)
Postgraduate	28.4 (432)	28.1 (254)
Hospital of recruitment, % (*n*)		
Bellevue	14.0 (219)	16.6 (174)
NYU–Brooklyn	35.4 (552)	28.11 (294)
NYU–Manhattan	50.6 (789)	55.3 (578)

NYU CHES, New York University Children’s Health and Environment Study.

Multivariable associations between maternal characteristics and perceived stress during the COVID-19 pandemic are presented in Table 2. Hispanic women reported lower PSS-4 scores than non-Hispanic White women (*β* = −0.66 for Hispanic vs. Non-Hispanic White women, 95%CI: −1.06, −0.26). Higher resilience was also associated with lower perceived stress (*β* = −1.36 per unit increase in resiliency score, 95%CI: −1.57, −1.15). Women with at least some college education had lower stress compared to those with high school educational level or less (*β* = −0.54, 95%CI: −1.03, −0.05). In contrast, a prior history of depression (*β* = 0.76, 95%CI: 0.45, 1.07), higher pre-pandemic general stress (*β* = 0.23 per unit increase in general stress score, 95%CI: 0.17, 0.29), and having had COVID-19 (*β* = 0.58, 95%CI: 0.23, 0.94) were associated with higher PSS-4 scores. We also observed a positive dose-response relation between current financial insecurity and perceived stress (Table 2). Additional adjustment for social support in a subgroup of women with this information at baseline assessment (*n* = 1213) minimally influenced the associations described above (data not shown), except that the association of Non-Hispanic Black race/ethnicity with PSS-4 score became statistically significant. Each unit increase in social support score was associated with a 0.04 point decrease in PSS-4 score (95%CI: −0.07, −0.01).

**TABLE 2:** Associations between maternal characteristics and current perceived stress score from a multivariable linear regression model (New York University Children’s Health and Environment Study, United States, 2020).

	Perceived stress score	
	*β*	(95% confidence interval)
Age at enrollment in the cohort, year	0.002	(−0.02, 0.03)
Assessment before June 1, 2021	0.25	(−0.06, 0.56)
Race/ethnicity		
Hispanic	−0.66	(−1.06, −0.26)
Non-Hispanic White	Ref	Ref
Non-Hispanic Black	−0.40	(−1.07, 0.28)
Non-Hispanic Asian	−0.38	(−0.85, 0.10)
Other/Multiple	−0.02	(−0.79, 0.75)
Education		
High school or less	Ref	Ref
Some college	−0.54	(−1.03, −0.05)
Bachelor’s degree	−0.24	(−0.76, 0.27)
Postgraduate	−0.07	(−0.43, 0.29)
Single (vs. married/partnered)	−0.03	(−0.46, 0.40)
Depression history (yes vs. no)^a^	0.75	(0.44, 1.06)
Current financial security		
Comfortable with extra	Ref	Ref
Enough but no extra	0.18	(−0.19, 0.55)
Have to cut back	0.81	(0.41, 1.21)
Cannot make ends meet	0.91	(0.41, 1.41)
Pre-pandemic general stress score^b^	0.22	(0.17, 0.28)
Brief resilience scale score^c^	−1.36	(−1.57, −1.15)
Currently pregnant (yes vs. no)	−0.10	(−0.48, 0.28)
Number of children in the household	0.07	(−0.08, 0.21)
Has been a COVID-19 case (yes vs. no)	0.58	(0.23, 0.94)
Had a child with COVID-19 (yes vs. no)	0.31	(−0.15, 0.77)

a Ever-depressed assessed *via* Patient Health Questionnaire-9 score (≥10) during pregnancy and/or Edinburgh Postnatal Depression Scale score (≥10) 4–12 months postpartum.

b Measured using instrument adapted from the American Psychological Association’s Stress in America survey (range = 1–10) with higher scores denoting higher stress.

c Measured using the Brief Resilience Scale (range = 1–5) with higher scores denoting greater resilience.

We observed that those with high stress were more likely to have been recruited from NYU Manhattan (which typically serves a greater proportion of affluent, non-Hispanic White women) and those with low stress were more likely to have been recruited from NYU Brooklyn (serving largely low-income Hispanic communities). As such, we reran the analysis using GEE with clustering on study sites. Results remained essentially unchanged (Supplementary Table S4), except that the association between having a child with COVID-19 and perceived stress became statistically significant (*β* = 0.38, 95%: 0.09, 0.67). Restricting the analysis to women who resided in the NYC metropolitan area who answered to the survey prior to 1 June 2020 yielded the same results, except that current financial insecurity was no longer associated with PSS-4 score (Supplementary Table S5). Also, the associations did not materially change when analysis was stratified by baseline financial security (Supplementary Table S6).

**FIGURE 1:**
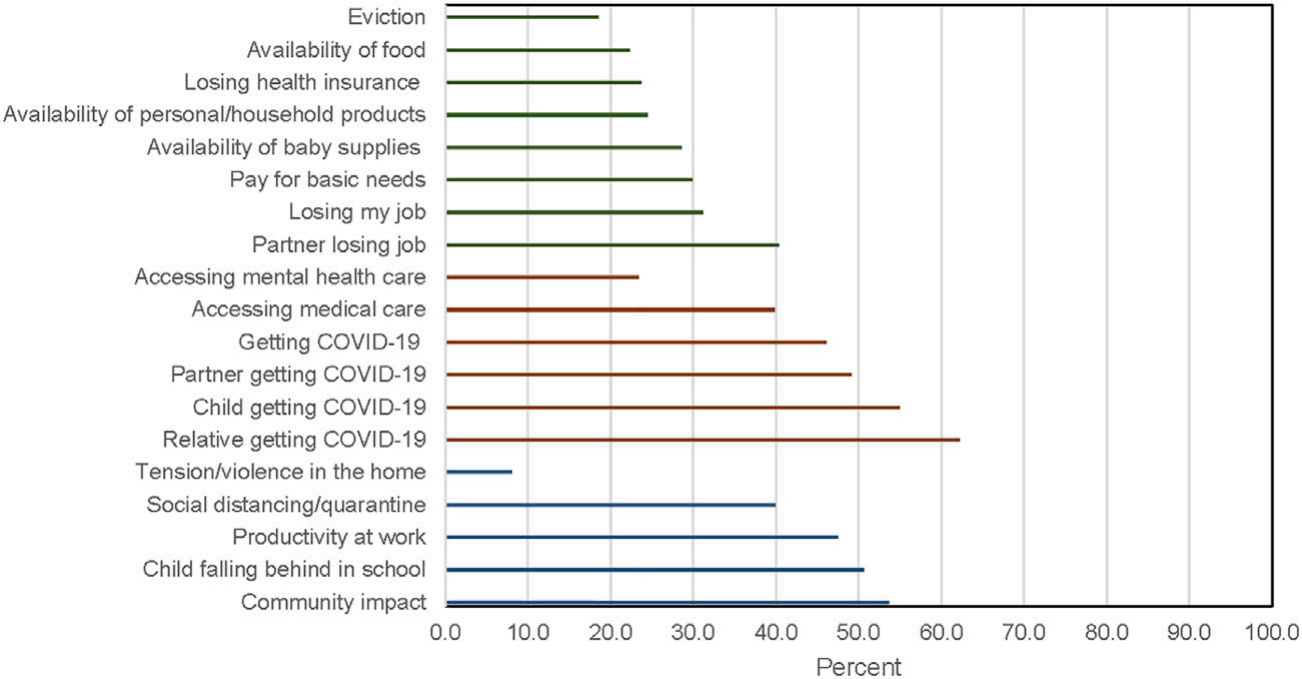
Percent women who reported intense concern for financial (green), health (red), and societal/familial (blue) factors following the outbreak of COVID-19, New York University Children’s Health and Environment Study (New York City, United States, April–August 2020).

Financial, health, and societal/familial concerns related to the COVID-19 pandemic were common (Figure 1). Health-related concerns were most frequent, followed by familial/societal and financial concerns. Domain scores were moderately correlated with each other (*ρ* = 0.43–0.59). In the models mutually adjusted for all three domain scores, health-related concerns were not associated with PSS-4 score (Table 3), but financial and familial/social COVID-related concerns were (β per unit increase in financial score = 0.33, 95%CI: 0.16, 0.50; and β per unit increase in familial/societal score = 0.35, 95%CI: 0.17, 0.54).

**TABLE 3:** Associations between COVID-19 concern domain scores and current perceived stress score (New York University Children’s Health and Environment Study, United States, 2020).

	PSS-4 score^b^	
	*β*	95% confidence interval
Financial COVID-19 concerns score^a^	0.33	0.16, 0.50
Health COVID-19 concerns score^a^	−0.02	−0.18, 0.13
Familial/societal COVID-19 concerns score^a^	0.35	0.17, 0.54

Models were adjusted for age, race/ethnicity, education, depression history, baseline financial security, pre-pandemic general stress levels, and resiliency score as well as marital status, number of children in the household, and date of assessment.

a COVID-19, concern domain scores are average scores (range = 0–4) with greater values indicating greater concern.

b PSS-4, Perceived Stress Score-4 items (range = 0–16).

Changes to prenatal care during the early stage of the pandemic were common among pregnant women or those who had recently delivered (Table 4). In particular, almost half of the pregnant women reported that the format of their prenatal care changed (e.g., from in-person to phone or telemedicine/video appointments); however, only 22.3% of women who recently delivered reported this, likely reflecting the fact that they were already in the late stage of pregnancy when the outbreak occurred. In contrast, changes in birth plan were generally uncommon and mainly limited to that fact that the intended support person(s) could not attend delivery. Among women who had recently delivered, major disruptions to their postnatal experiences included lack of support (e.g., family and friends not able to visit (68.9%), family and friends not able to help after baby was born (56.3%), or being sent home early from the hospital (39.8%)). The COVID-19 outbreak affected newborn feeding plans in a limited number of women (*n* = 17, 16.5%).

**TABLE 4:** Changes to prenatal care, birth plan, postnatal care/experiences, and childcare (New York University Children’s Health and Environment Study, United States, 2020).

	Currently pregnant (*n* = 228)^a^		Recently delivered (*n* = 103)^a^		Mothers of young children in childcare before COVID-19 (*n* = 531)^a^	
	N	%	N	%	N	%
Prenatal care						
No change	52	22.8	48	46.6		
Care has improved	10	4.4	8	7.8		
Care has worsened	24	10.5	7	6.8		
Changed prenatal healthcare provider(s)	22	9.7	3	2.9		
Have not gone to prenatal appointments due to concern about entering my healthcare provider’s office	31	13.6	12	11.7		
Healthcare provider has cancelled or reduced frequency of my prenatal visit(s)	62	27.2	24	23.3		
Have had more prenatal visits	7	3.1	2	1.9		
Format of prenatal care has changed (e.g., from in-person to phone or telemedicine/video appointments)	113	49.6	23	22.3		
Healthcare provider has directed me to self-isolate or quarantine	5	2.2	2	1.9		
Birth plan						
No change	161	70.6	68	66.0		
Changing/changed hospitals	18	7.9	2	1.9		
Changing/changed from hospital delivery to home birth	3	1.3	1	1.0		
Will be/was induced because of COVID-19 infection	0	0.0	1	1.0		
Will have/had C-section because of COVID-19 infection	1	0.4	1	1.0		
Planned C-section or labor induction is being/was changed	0	0.0	2	1.9		
Intended support person(s) will not be/were not permitted to attend delivery	41	18.0	26	25.2		
Experience following birth						
Separated from baby immediately after delivery			13	12.6		
Had to pump breastmilk and have someone else bottle-feed baby			7	6.8		
Changed from planning to breastfeed to feeding only formula			9	8.7		
Changed from planning to feed only formula to breastfeeding			1	1.0		
Family and friends not able to visit			71	68.9		
Sent home early from the hospital			41	39.8		
Postnatal visits cancelled or postponed			25	24.3		
Family and friends not able to help after baby was born			58	56.3		
None of the above			10	9.7		
Regular childcare, daycare, or preschool						
Had difficulty arranging for childcare					87	16.4
Had to pay more for childcare					23	4.3
My partner or I had to change our work schedule to care for our child (ren) ourselves					232	43.7
My child’s daycare/preschool closed completely because of the COVID-19 outbreak					348	65.5
My child’s daycare/preschool is open only for children of essential workers					38	7.2
My child had to change to a different daycare/preschool					9	1.7
My child’s daycare/preschool is offering online learning					129	24.3
My regular childcare/daycare has not been affected by the COVID-19 outbreak					26	4.9

^a^Currently pregnant and recently delivered groups are mutually exclusive but each are not mutually exclusive with mothers of young children.

Among women who had a child in childcare, daycare, or preschool (*n* = 531), disruptions were extremely common, with less than 5% reporting no change in their childcare arrangement (Table 4). Forty-three percent of women or their partners had to change their work schedule to accommodate the change in childcare or preschool during early stages of the pandemic in NYC. Childcare or preschool disruptions were independently associated with perceived stress in the subset of women with young children in childcare or preschool, even after adjusting for all demographic, stress, and psychological covariates (β per additional problem = 0.30, 95% CI: 0.08, 0.53).

## Discussion

Among pregnant women and/or mothers of young children in a well-characterized and sociodemographically diverse NYC cohort, a history of depression, current financial difficulties, and being infected with COVID-19 were associated with higher perceived stress. In contrast, being Hispanic and having higher resiliency scores, higher educational levels, and preexisting social support were protective against high perceived stress. The effect sizes were the largest for resilience score and being Hispanic. Major contributors to current stress were financial and familial/societal factors related to the COVID-19 pandemic. Among pregnant women, changes to prenatal care were common, as were changes to experiences following birth among postpartum women and changes in childcare among mothers of young children.

The COVID-19 pandemic can act as a traumatic stressor in individuals [13], but it remains unclear whether pre-pandemic mental health and sociodemographic characteristics might increase susceptibility to stress during the pandemic. Several studies have examined mental health consequences of this pandemic in the general adult population using a longitudinal design [35, 36]. One systematic review of studies of mental health outcomes in pregnant women that was published 2021 showed high levels of psychological symptoms but also highlighted that the majority of these studies examined predictors and correlates in a cross-sectional design [32]. In the present study, we examined sociodemographic characteristics and predictors of stress in pregnant women and mothers of young children in the context of this pandemic. While we did not have direct measures of post-outbreak mental health outcomes, we examined the role of pre-pandemic mental health on measures of current perceived stress. Our results highlighting the importance of individual characteristics such as education, resiliency, and financial security are novel and consistent with some other reports in non-pregnant and pregnant populations [37, 38]. In contrast to others studies [32], maternal age and number of children in the household (as a proxy measure of household size) were not associated with higher perceived stress, probably explained by the narrow age range and the fact that the cohort consisted of either nulliparous women. In terms of race/ethnicity, we found that non-Hispanic White women had the highest perceived stress compared with Hispanic, non-Hispanic Black, and non-Hispanic Asian women. The associations were robust when we controlled for social support. While these findings should be interpreted with caution considering the particularly small number of non-Hispanic Black women in this cohort (70 women, 4.5%), they came as a surprise and are different from reports showing that communities of color have been disproportionately affected by the COVID-19 pandemic [39]. National studies have reported higher mental health and stress burdens among non-White individuals [40–42]. In contrast, another COVID-19 study representative of United States (US) adults previously showed that White adults were more likely to report stress and worry about the health of their families as well as feeling isolated and alone compared with Black and Hispanic adults [40]. Conversely, McKnight-Eily et al. reported that Hispanic adults were the most likely to have depressive symptoms, suicidal thoughts, substance use initiation, and worries about having enough food and stable housing [40]. Medium- and long-term follow-up studies later in or after the pandemic will clarify whether higher perceived stress will translate into higher rates of mental health problems in racial and ethnic subgroups.

When we accounted for financial- and familial/societal-related concerns, health-related concerns were not among the main drivers of overall perceived stress. The null finding with regard to health-related concerns is in contrast with earlier cross-sectional reports that fear of COVID-19 infection was associated with pregnancy-related anxiety and stress [5, 43]. A systematic review of 31 studies on anxiety and depressive symptoms in pregnant women showed that fear of contagion and concerns regarding the health of the fetus were major predictors of mental health in these women [44]. But several of these earlier studies did not simultaneously examine other sources of stress, such as financial difficulties. One of the studies was conducted in Italy, which has less social inequality compared with the US, and the other was a cross-sectional survey on social media, which did not capture the socioeconomic diversity of the US. Within the domain of familial/societal-related concerns, one of the items we included in the survey, i.e., some level of concern regarding increasing tension and/or domestic violence in the home, endorsed by nearly 30% of women in our study, has been proposed as an important and common predictor of stress in women in the US during the pandemic [45].

Women in our cohort reported significant changes in prenatal care, postpartum experiences, and childcare arrangements following the outbreak, with pregnant women reporting the highest stress levels. Other studies have reported that pregnancy-related anxiety increased during early stages of the pandemic, leading to increases in depressive symptoms [15, 43]. This stress burden among pregnant women was found to be driven by community- and individual-level factors related to socioeconomic inequality such as living in communities with lower education levels and fewer English speakers [15]. Implications of these observations are two-fold. First, considering the source of stress in pregnant women and mothers of young children, targeted interventions such as financial aid to families with children might alleviate the mental health burden in women. Second, heightened stress and subsequent mental health impacts on women during the peri-partum period may influence physical and mental well-being of their offspring. Future follow-up studies are needed to assess child outcomes of maternal stress during the pandemic and determine the need for support systems in future similar events.

Our study leveraged a well-characterized cohort with prospectively collected pre-COVID data. This is a unique attribute among studies that examine the aftermath of traumatic exposures, as they usually lack data on conditions before the event and rely on retrospective reports [46]. The cohort had substantial racial/ethnic as well as socioeconomic diversity, which allowed for examination of effects across multiple demographic subgroups and investigation of specific COVID-related concerns, which were absent in prior literature. Our study also benefited from data collection that began in April 2020 in NYC, which was at the height of the initial wave of the outbreak. However, our study was also subject to limitations. First, we relied on women’s self-report of perceived stress and did not have objective assessments of stress or diagnoses of anxiety, depression, or clinically relevant outcomes such as drug use or suicidality. Second, despite inclusion of important pre-pandemic data (e.g., mental health and social support), we had to rely on retrospective recall for certain measures, such as pre-COVID general stress level or resiliency, which were not readily available in the cohort. Our survey was conducted during the early stage of the pandemic and persistent elevated stress levels cannot be inferred from these data. Also, this analysis was in response to an unprecedented event and we did not perform a power calculation before implementing the questionnaire or performing the analysis. Following recommendations [47, 48], we do not present a post-hoc power calculation for interpretation of the results.

Within a diverse sample of pregnant women and/or mothers of young children, our findings underscore the importance of financial- and familial/societal-related concerns as major sources of stress during the pandemic. Additional risk factors of higher stress during the pandemic were similar to those reported for other major traumatic events, such as prior history of depression and lower resiliency. Since disruptions in prenatal care and interruption of childcare arrangements were common among participating women, follow-up studies are needed to monitor the potential for long-term stress and mental health effects in mothers and subsequent impacts in their offspring.
