# Supplementary material for: Maternal Perceived Stress During the COVID-19 Pandemic: Pre-Existing Risk Factors and Concurrent Correlates in New York City Women
Source: Int J Public Health. 2022 Apr 11;67:1604497. doi: 10.3389/ijph.2022.1604497 (PMC9035490; doi:10.3389/ijph.2022.1604497)
Supplement: Supplementary file 1 [file DataSheet1.docx]

Supplementary Material for the manuscript “**Maternal perceived stress during the COVID-19 pandemic: pre-existing risk factors and concurrent correlates in New York City women**”

Akhgar Ghassabian, Melanie H. Jacobson, Linda G. Kahn^,^ Sara G. Brubaker, Shilpi S. Mehta-Lee, Leonardo Trasande

**Supplemental Table 1**. Concerns in relation to the COVID-19 epidemic, New York University Children’s Health and Environment Study (NYU CHES), New York, April-August, 2020.

**Supplementary Table 2.** Participant baseline characteristics by stress level early in the COVID-19 pandemic, New York University Children’s Health and Environment Study (NYU CHES), New York, April-August, 2020.

**Supplementary Table 3**. Participant characteristics during the early stage of the COVID-19 pandemic by concurrent stress levels, New York University Children’s Health and Environment Study (NYU CHES), New York, April-August, 2020.

**Supplementary Table 4.** Associations between maternal characteristics and current perceived stress score from a multivariable linear regression model using generalized estimating equation (GEE) with sites as the cluster, New York, April-August 2020.

**Supplementary Table 5.** Associations between maternal characteristics and current perceived stress scores, restricted to women living in the New York City Metropolitan Area and those who took the questionnaire before June 1, 2020 (n=1082).

**Supplemental Table 1**. Concerns in relation to the COVID-19 epidemic, New York University Children’s Health and Environment Study (NYU CHES), New York, April-August, 2020.

| Concern | Domain | | | N | % |
| --- | --- | --- | --- | --- | --- |
| ***Impact on the community*** | Societal/familial | | |  |  |
| Not concerned at all |  | | | 149 | 10.1 |
| Slightly concerned |  | | | 179 | 12.2 |
| Somewhat concerned |  | | | 354 | 24.1 |
| Moderately concerned |  | | | 344 | 23.4 |
| Extremely concerned |  | | | 446 | 30.3 |
| N/A |  |  |  | 36 |  |
| ***My child will fall behind in school*** | Societal/familial | | | | |
| Not concerned at all |  | | | 168 | 20.5 |
| Slightly concerned |  | | | 101 | 12.3 |
| Somewhat concerned |  | | | 135 | 16.5 |
| Moderately concerned |  | | | 146 | 17.8 |
| Extremely concerned |  | | | 269 | 32.8 |
| N/A |  |  |  | 695 |  |
| ***Not being productive at work because of having to supervise my child's homeschooling*** | Societal/familial | | | | |
| Not concerned at all |  | | | 174 | 22.1 |
| Slightly concerned |  | | | 98 | 12.4 |
| Somewhat concerned |  | | | 142 | 18.0 |
| Moderately concerned |  | | | 150 | 19.0 |
| Extremely concerned |  | | | 225 | 28.5 |
| N/A |  |  |  | 718 |  |
| ***Social distancing/ quarantine*** | Societal/familial | | |  |  |
| Not concerned at all |  | | | 225 | 14.9 |
| Slightly concerned |  | | | 286 | 19.0 |
| Somewhat concerned |  | | | 394 | 26.1 |
| Moderately concerned |  | | | 336 | 22.3 |
| Extremely concerned |  | | | 266 | 17.7 |
| N/A |  |  |  | 53 |  |
| ***Increasing tension and/or violence in the home*** | Societal/familial | | | | |
| Not concerned at all |  | | | 942 | 71.9 |
| Slightly concerned |  | | | 163 | 12.4 |
| Somewhat concerned |  | | | 100 | 7.6 |
| Moderately concerned |  | | | 60 | 4.6 |
| Extremely concerned |  | | | 45 | 3.4 |
| N/A |  |  |  | 202 |  |
| ***An elderly relative or close family friend will get COVID-19*** | Health | | | | |
| Not concerned at all |  | | | 156 | 11.1 |
| Slightly concerned |  | | | 151 | 10.8 |
| Somewhat concerned |  | | | 224 | 16.0 |
| Moderately concerned |  | | | 303 | 21.6 |
| Extremely concerned |  | | | 570 | 40.6 |
| N/A |  |  |  | 94 |  |
| ***My unborn child will be exposed to COVID-19 or my child/children will get COVID-19*** | Health | | | | |
| Not concerned at all |  | | | 158 | 10.8 |
| Slightly concerned |  | | | 244 | 16.7 |
| Somewhat concerned |  | | | 255 | 17.5 |
| Moderately concerned |  | | | 294 | 20.2 |
| Extremely concerned |  | | | 507 | 34.8 |
| N/A |  |  |  | 102 |  |
| ***My partner will get COVID-19*** | Health | | |  |  |
| Not concerned at all |  | | | 185 | 14.1 |
| Slightly concerned |  | | | 229 | 17.4 |
| Somewhat concerned |  | | | 255 | 19.4 |
| Moderately concerned |  | | | 269 | 20.5 |
| Extremely concerned |  | | | 375 | 28.6 |
| N/A |  |  |  | 177 |  |
| ***I will get COVID-19*** | Health | | |  |  |
| Not concerned at all |  | | | 197 | 14.2 |
| Slightly concerned |  | | | 246 | 17.7 |
| Somewhat concerned |  | | | 305 | 22.0 |
| Moderately concerned |  | | | 270 | 19.5 |
| Extremely concerned |  | | | 370 | 26.7 |
| N/A |  |  |  | 112 |  |
| ***Not being able to access medical care for myself or my family*** | Health | | | | |
| Not concerned at all |  | | | 364 | 24.5 |
| Slightly concerned |  | | | 241 | 16.2 |
| Somewhat concerned |  | | | 289 | 19.5 |
| Moderately concerned |  | | | 245 | 16.5 |
| Extremely concerned |  | | | 346 | 23.3 |
| N/A |  |  |  | 29 |  |
| ***Not being able to access mental health care for myself or my family*** | Health | | | | |
| Not concerned at all |  | | | 638 | 46.0 |
| Slightly concerned |  | | | 223 | 16.1 |
| Somewhat concerned |  | | | 203 | 14.6 |
| Moderately concerned |  | | | 142 | 10.2 |
| Extremely concerned |  | | | 182 | 13.1 |
| N/A |  |  |  | 101 |  |
| ***My partner or someone I depend upon for income losing their job*** | Financial | | | | |
| Not concerned at all |  | | | 373 | 26.8 |
| Slightly concerned |  | | | 245 | 17.6 |
| Somewhat concerned |  | | | 213 | 15.3 |
| Moderately concerned |  | | | 193 | 13.9 |
| Extremely concerned |  | | | 368 | 26.4 |
| N/A |  |  |  | 111 |  |
| ***Losing my job*** | Financial | | |  |  |
| Not concerned at all |  |  |  | 363 | 34.1 |
| Slightly concerned |  |  |  | 207 | 19.4 |
| Somewhat concerned |  |  |  | 164 | 15.4 |
| Moderately concerned |  |  |  | 145 | 13.6 |
| Extremely concerned |  |  |  | 187 | 17.5 |
| N/A |  |  |  | 428 |  |
| ***Not being able to pay for basic needs (rent/mortgage, food, medicine, baby supplies, etc.)*** | Financial | | | | |
| Not concerned at all |  | | | 504 | 33.0 |
| Slightly concerned |  | | | 274 | 17.9 |
| Somewhat concerned |  | | | 294 | 19.2 |
| Moderately concerned |  | | | 198 | 13.0 |
| Extremely concerned |  | | | 258 | 16.9 |
| N/A |  |  |  | 16 |  |
| ***Availability of baby supplies (e.g., formula, diapers, wipes)*** | Financial | | | | |
| Not concerned at all |  | | | 437 | 29.5 |
| Slightly concerned |  | | | 319 | 21.5 |
| Somewhat concerned |  | | | 302 | 20.4 |
| Moderately concerned |  | | | 218 | 14.7 |
| Extremely concerned |  | | | 205 | 13.8 |
| N/A |  |  |  | 55 | 3.6 |
| ***Availability of personal care products or household supplies*** | Financial | | | | |
| Not concerned at all |  | | | 467 | 30.9 |
| Slightly concerned |  | | | 346 | 22.9 |
| Somewhat concerned |  | | | 330 | 21.8 |
| Moderately concerned |  | | | 225 | 14.9 |
| Extremely concerned |  | | | 144 | 9.5 |
| N/A |  |  |  | 22 |  |
| ***Loss of health insurance*** | Financial | | |  |  |
| Not concerned at all |  | | | 606 | 47.1 |
| Slightly concerned |  | | | 223 | 17.3 |
| Somewhat concerned |  | | | 152 | 11.8 |
| Moderately concerned |  | | | 121 | 9.4 |
| Extremely concerned |  | | | 184 | 14.3 |
| N/A |  |  |  | 220 |  |
| ***Availability of food*** | Financial | | |  |  |
| Not concerned at all |  | | | 540 | 35.4 |
| Slightly concerned |  | | | 346 | 22.7 |
| Somewhat concerned |  | | | 299 | 19.6 |
| Moderately concerned |  | | | 217 | 14.2 |
| Extremely concerned |  | | | 122 | 8.0 |
| N/A |  |  |  | 10 |  |
| ***Being evicted*** | Financial | | |  |  |
| Not concerned at all |  | | | 779 | 58.7 |
| Slightly concerned |  | | | 157 | 11.8 |
| Somewhat concerned |  | | | 146 | 11.0 |
| Moderately concerned |  | | | 112 | 8.4 |
| Extremely concerned |  | | | 133 | 10.0 |
| N/A |  |  |  | 176 |  |

| **Supplementary Table 2.** Participant baseline characteristics by stress level early in the COVID-19 pandemic, New York University Children’s Health and Environment Study (NYU CHES), New York, April-August, 2020. | | | | | |  |
| --- | --- | --- | --- | --- | --- | --- |
|  | **Overall** | **Low stress** | **Moderate stress** | **High stress** |  | |
|  | N=1560 | N=408, 26.2% | n=859, 55.1% | N=291, 18.7% | p | |
| Age, mean (SD) | 32.1 (5.6) | 31.5 (5.6) | 32.5 (5.6) | 31.8 (5.3) | <0.01 | |
| Race/ethnicity, n (%) |  |  |  |  | 0.06 | |
| Hispanic | 781 (50.2) | 218 (53.6) | 419 (48.9) | 142 (49.0) |  | |
| White | 518 (33.3) | 120 (29.5) | 283 (33.0) | 115 (39.7) |  | |
| Black | 70 (4.5) | 20 (4.9) | 39 (4.6) | 11 (3.8) |  | |
| Asian | 141 (9.1) | 40 (9.8) | 85 (9.9) | 16 (5.5) |  | |
| Other/Multiple | 46 (3.0) | 9 (2.2) | 31 (3.6) | 6 (2.1) |  | |
| Recruitment site, n (%) |  |  |  |  | 0.02 | |
| Bellevue | 219 (14.0) | 47 (11.5) | 129 (15.0) | 42 (14.4) |  | |
| NYU–Brooklyn | 552 (35.4) | 172 (42.2) | 287 (33.4) | 93 (32.0) |  | |
| NYU–Manhattan | 789 (50.6) | 189 (46.3) | 443 (51.6) | 156 (53.6) |  | |
| Public insurance, n (%) | 770 (50.3) | 215 (53.9) | 421 (49.7) | 133 (47.0) | 0.18 | |
| Annual household income, n (%) |  |  |  |  | 0.27 | |
| <$30,000 | 272 (17.4) | 72 (17.7) | 145 (16.9) | 55 (18.9) |  | |
| $30,000 to $100,000 | 270 (17.3) | 67 (16.4) | 143 (16.7) | 60 (20.6) |  | |
| ≥$100,000 | 601 (38.5) | 146 (35.8) | 345 (40.2) | 109 (37.5) |  | |
| Don't know | 417 (26.7) | 123 (30.2) | 226 (26.3) | 67 (23.0) |  | |
| Education, n (%) |  |  |  |  | 0.63 | |
| High school or less | 504 (33.1) | 144 (36.0) | 273 (32.6) | 86 (30.2) |  | |
| Some college | 223 (14.6) | 57 (14.3) | 119 (14.2) | 46 (16.1) |  | |
| Bachelor's degree | 365 (24.0) | 93 (23.3) | 208 (24.9) | 64 (22.5) |  | |
| Postgraduate | 432 (28.4) | 106 (26.5) | 237 (28.3) | 89 (31.2) |  | |
| Married/partnered, n (%) | 1354 (88.9) | 362 (90.3) | 732 (87.7) | 258 (90.2) | 0.28 | |
| Depression history, n (%)^a^ | 358 (23.4) | 54 (13.4) | 194 (23.1) | 108 (37.9) | <0.01 | |
| Employed, n (%) | 992 (65.3) | 258 (65.0) | 539 (64.6) | 193 (67.5) | 0.68 | |
| Social support score, median (IQR)^b^ | 28 (6) | 29 (5) | 28 (6) | 27 (7) | <0.01 | |

^a^ Ever-depressed via Patient Health Questionnaire-9 score (≥10) during pregnancy and/or Edinburgh Postnatal Depression Scale score (≥10) 4-12 months postpartum

^b^ Measured using the ENRICHD Social Support Instrument (range= 6-30) among 1249 mothers who completed this in the second trimester

IQR: interquartile range; NYU CHES: New York University Children’s Health and Environment Study

| **Supplementary Table 3**. Participant characteristics during the early stage of the COVID-19 pandemic by concurrent stress levels, New York University Children’s Health and Environment Study (NYU CHES), New York, April-August, 2020. | | | | | |
| --- | --- | --- | --- | --- | --- |
|  | **Overall** | **Low stress** | **Moderate stress** | **High stress** |  |
|  | N=1560 | N=408, 26.2% | n=859, 55.1% | N=291, 18.7% | *P* |
| Calendar time of assessment, n (%) |  |  |  |  | 0.78 |
| April-May 2020 | 1222 (78.3) | 315 (77.2) | 678 (78.9) | 228 (78.4) |  |
| June-August 2020 | 338 (21.7) | 93 (22.8) | 181 (21.1) | 63 (21.7) |  |
| Living in NYC metro area at time of assessment, n (%) | 1370 (87.8) | 348 (85.3) | 765 (89.1) | 257 (88.3) | 0.15 |
| Pre-COVID financial security, n (%) |  |  |  |  | 0.02 |
| Comfortable with extra | 624 (40.4) | 193 (47.5) | 328 (38.5) | 103 (36.1) |  |
| Enough but no extra | 669 (43.3) | 156 (38.4) | 387 (45.4) | 126 (44.2) |  |
| Have to cut back | 166 (10.8) | 41 (10.1) | 86 (10.1) | 39 (13.7) |  |
| Cannot make ends meet | 85 (5.5) | 16 (3.9) | 51 (6.0) | 17 (6.0) |  |
| Current financial security, n (%) |  |  |  |  | <0.01 |
| Comfortable with extra | 392 (25.3) | 125 (30.8) | 209 (24.5) | 58 (20.2) |  |
| Enough but no extra | 428 (27.7) | 120 (29.6) | 245 (28.7) | 63 (22.0) |  |
| Have to cut back | 496 (32.1) | 121 (29.8) | 273 (32.0) | 102 (35.5) |  |
| Cannot make ends meet | 231 (14.9) | 40 (9.9) | 126 (14.8) | 64 (22.3) |  |
| ∆ Financial security, mean (SD)^a^ | 0.6 (0.8) | 0.5 (0.8) | 0.5 (0.8) | 0.7 (0.9) | <0.01 |
| Pre-COVID general stress score, mean (SD)^b^ | 3.8 (2.3) | 3.0 (2.1) | 3.8 (2.2) | 4.7 (2.3) | <0.01 |
| Brief resilience scale score, mean (SD)^c^ | 3.4 (0.7) | 3.8 (0.6) | 3.4 (0.6) | 3.1 (0.7) | <0.01 |
| Children living in house, mean (SD) | 1.7 (1.1) | 1.7 (1.1) | 1.8 (1.1) | 1.8 (1.1) | 0.38 |
| Pregnant, n (%) | 228 (14.7) | 75 (18.5) | 119 (14.0) | 34 (11.7) | 0.03 |
| COVID-19 case (self), n (%)^d^ | 283 (18.1) | 51 (12.5) | 149 (17.4) | 83 (28.5) | <0.01 |
| COVID-19 case (child living in household), n (%)^d^ | 149 (9.7) | 30 (7.4) | 72 (8.5) | 47 (16.3) | <0.01 |
| COVID-19 hospitalization in household, n (%) | 14 (0.9) | 2 (0.5) | 6 (0.7) | 6 (2.1) | 0.09 |
| Childcare problem score, mean (SD)^f^ | 1.6 (0.9) | 1.5 (0.9) | 1.6 (0.9) | 1.8 (1.1) | <0.01 |

^a^ Higher numbers denote a greater loss of financial security

^b^ Measured using instrument adapted from the American Psychological Association’s Stress in America survey (range=1-10) with higher scores denoting higher stress

^c^ Measured using the Brief Resilience Scale (range=1-5) with higher scores denoting greater resilience

^d^ COVID-19 case defined as meeting WHO criteria, healthcare practitioner diagnosis, or positive polymerase chain reaction or antibody testing

^e^ Among 531 mothers of young children enrolled in childcare before COVID-19, sum of seven yes/no questions about difficulties with childcare

**Supplementary Table 4.** Associations between maternal characteristics and current perceived stress score from a multivariable linear regression model using generalized estimating equation (GEE) with sites as the cluster, New York, April-August 2020.

|  | Perceived Stress Score | |
| --- | --- | --- |
|  | β | (95% Confidence Interval) |
| Age at enrollment in the cohort, year | 0.01 | (0.00, 0.02) |
| Race/ethnicity |  |  |
| Hispanic | -0.85 | (-1.12, -0.59) |
| Non-Hispanic White | Ref | Ref |
| Non-Hispanic Black | -0.42 | (-1.05, 0.21) |
| Non-Hispanic Asian | -0.37 | (-0.86, 0.11) |
| Other/Multiple | -0.01 | (-0.47, 0.46) |
| Education |  |  |
| High school or less | Ref | Ref |
| Some college | -0.54 | (-1.03, -0.05) |
| Bachelor's degree | -0.24 | (-0.76, 0.27) |
| Postgraduate | -0.07 | (-0.43, 0.29) |
| Single (vs. married/partnered) | -0.03 | (-0.46, 0.40) |
| Depression history (yes vs. no)^a^ | 0.75 | (0.44, 1.06) |
| Current financial security |  |  |
| Comfortable with extra | Ref | Ref |
| Enough but no extra | 0.18 | (-0.19, 0.55) |
| Have to cut back | 0.81 | (0.41, 1.21) |
| Cannot make ends meet | 0.91 | (0.41, 1.41) |
| Pre-pandemic general stress score^b^ | 0.22 | (0.17, 0.28) |
| Brief resilience scale score^c^ | -1.36 | (-1.57, -1.15) |
| Currently pregnant (yes vs. no) | -0.10 | (-0.48, 0.28) |
| Number of children in the household | 0.07 | (-0.08, 0.21) |
| Has been a COVID-19 case (yes vs. no) | 0.58 | (0.23, 0.94) |
| Had a child with COVID-19 (yes vs. no) | 0.31 | (-0.15, 0.77) |

^a^ Ever-depressed assessed via Patient Health Questionnaire-9 score (≥10) during pregnancy and/or Edinburgh Postnatal Depression Scale score (≥10) 4-12 months postpartum

^b^ Measured using instrument adapted from the American Psychological Association’s Stress in America survey (range=1-10) with higher scores denoting higher stress

^c^ Measured using the Brief Resilience Scale (range=1-5) with higher scores denoting greater resilience

**Supplementary Table 5.** Associations between maternal characteristics and current perceived stress scores, restricted to women living in the New York City Metropolitan Area and those who took the questionnaire before June 1, 2020 (n=1082).

|  | Perceived Stress Score | |
| --- | --- | --- |
|  | β | (95% Confidence Interval) |
| Age at enrollment in the cohort, year | -0.002 | (-0.03, 0.02) |
| Race/ethnicity |  |  |
| Hispanic | -0.50 | (-0.96, -0.34) |
| Non-Hispanic White | Ref | Ref |
| Non-Hispanic Black | -0.48 | (-1.27, 0.32) |
| Non-Hispanic Asian | -0.23 | (-0.79, 0.34) |
| Other/Multiple | -0.53 | (-1.49, 0.43) |
| Education |  |  |
| High school or less | Ref | Ref |
| Some college | -0.54 | (-1.11, 0.03) |
| Bachelor's degree | -0.09 | (-0.70, 0.51) |
| Postgraduate | -0.12 | (-0.56, 0.31) |
| Single (vs. married/partnered) | -0.01 | (-0.51, 0.50) |
| Depression history (yes vs. no)^a^ | 0.67 | (0.31, 1.03) |
| Current financial security |  |  |
| Comfortable with extra | Ref | Ref |
| Enough but no extra | 0.04 | (-0.40, 0.49) |
| Have to cut back | 0.54 | (0.07, 1.01) |
| Cannot make ends meet | 0.61 | (0.05, 1.18) |
| Pre-pandemic general stress score^b^ | 0.21 | (0.14, 0.28) |
| Brief resilience scale score^c^ | -1.42 | (-1.66, -1.18) |
| Currently pregnant (yes vs. no) | -0.21 | (-0.64, 0.22) |
| Number of children in the household | 0.07 | (-0.09, 0.23) |
| Has been a COVID-19 case (yes vs. no) | 0.59 | (0.18, 1.01) |
| Had a child with COVID-19 (yes vs. no) | 0.35 | (-0.18, 0.87) |

^a^ Ever-depressed assessed via Patient Health Questionnaire-9 score (≥10) during pregnancy and/or Edinburgh Postnatal Depression Scale score (≥10) 4-12 months postpartum

^b^ Measured using instrument adapted from the American Psychological Association’s Stress in America survey (range=1-10) with higher scores denoting higher stress

^c^ Measured using the Brief Resilience Scale (range=1-5) with higher scores denoting greater resilience

| **Supplemental Table 6.** Associations between maternal characteristics and current perceived stress score, stratified by baseline financial security. | | | | | | | | | |
| --- | --- | --- | --- | --- | --- | --- | --- | --- | --- |
|  | PSS-4 score^a^ | | | | | | | | |
|  | Comfortable with extra  (n=624) | | | Enough but no extra  (n=669) | | | Have to cut back or cannot make ends meet (n=251) | | |
|  | β | 95% Confidence Interval | | β | 95% Confidence Interval | | β | 95% Confidence Interval | |
| Age at enrollment in the cohort, year | 0.00 | -0.04, 0.04 | | 0.01 | -0.03, 0.04 | | 0.03 | -0.03, 0.08 | |
| Race/ethnicity |  |  |  |  |  |  |  |  |  |
| Hispanic | -0.99 | -1.53, -0.45 | | -0.78 | -1.26, -0.31 | | -0.12 | -1.35, 1.11 | |
| Non-Hispanic White | Ref | Ref | | Ref | Ref | | Ref | Ref | |
| Non-Hispanic Black | -1.10 | -2.19, -0.01 | | 0.25 | -0.72, 1.23 | | -0.84 | -2.67, 0.99 | |
| Non-Hispanic Asian | -0.48 | -1.11, 0.15 | | -0.21 | -0.97, 0.54 | | 0.05 | -1.94, 2.03 | |
| Other/Multiple | 0.06 | -0.98, 1.09 | | 0.02 | -1.24, 1.29 | | -0.65 | -3.38, 2.08 | |
| Depression history (yes vs. no)^b^ | 0.82 | 0.28, 1.36 | | 0.75 | 0.31, 1.19 | | 0.63 | -0.07, 1.34 | |
| Current financial security |  |  |  |  |  |  |  |  |  |
| Comfortable with extra | Ref | Ref | | Ref | Ref | | Ref | Ref | |
| Enough but no extra | 0.06 | -0.43, 0.56 | | -0.78 | -1.99, 0.44 | | -1.03 | -4.86, 2.80 | |
| Have to cut back | 1.26 | 0.61, 1.91 | | -0.44 | -1.66, 0.78 | | 0.05 | -3.45, 3.56 | |
| Cannot make ends meet | 0.46 | -0.76, 1.68 | | -0.21 | -1.51, 1.09 | | 0.38 | -3.14, 3.90 | |
| Pre-pandemic general stress score^c^ | 0.25 | 0.14, 0.37 | | 0.20 | 0.11, 0.28 | | 0.29 | 0.17, 0.41 | |
| Brief resilience scale score^d^ | -1.39 | -1.70, -1.08 | | -1.35 | -1.66, -1.04 | | -1.20 | -1.78, -0.63 | |
| Currently pregnant (yes vs. no) | -0.17 | -0.70, 0.36 | | -0.26 | -0.80, 0.29 | | 0.42 | -0.71, 1.54 | |
| Has been a COVID-19 case (yes vs. no) | 0.75 | 0.20, 1.30 | | 0.50 | -0.02, 1.02 | | 0.33 | -0.61, 1.27 | |
| Had a child with COVID-19 (yes vs. no) | 0.55 | -0.17, 1.27 | | 0.25 | -0.39, 0.88 | | 0.13 | -1.22, 1.48 | |
| ^a^ PSS-4: Perceived Stress Score – 4 items (range 0 -16)  ^b^ Ever-depressed assessed via Patient Health Questionnaire-9 score (≥10) during pregnancy and/or Edinburgh Postnatal Depression Scale score (≥10) 4-12 months postpartum  ^c^ Measured using instrument adapted from the American Psychological Association’s Stress in America survey (range=1-10) with higher scores denoting greater stress  ^d^ Measured using the Brief Resilience Scale (range=1-5) with higher scores denoting greater resilience | | | | | | | | | |
